# Aerobiology matters: Why people in the community access pollen information and how they use it

**DOI:** 10.1002/clt2.70031

**Published:** 2025-01-24

**Authors:** Danielle E. Medek, Constance H. Katelaris, Andelija Milic, Paul J. Beggs, Edwin R. Lampugnani, Don Vicendese, Bircan Erbas, Janet M. Davies

**Affiliations:** ^1^ School of Biomedical Sciences Centre Immunology and Infection Control Centre for Environment Queensland University of Technology Brisbane Queensland Australia; ^2^ School of Medicine Western Sydney University Sydney New South Wales Australia; ^3^ Department of Immunology Campbelltown Hospital Sydney New South Wales Australia; ^4^ Faculty of Science and Engineering School of Natural Sciences Macquarie University Sydney New South Wales Australia; ^5^ School of Biosciences The University of Melbourne Parkville Victoria Australia; ^6^ The Melbourne School of Population and Global Health University of Melbourne Parkville Victoria Australia; ^7^ School of Public Health LaTrobe University Bundoora Victoria Australia; ^8^ Present address: AirHealth Pty Ltd Parkville Victoria Australia; ^9^ Present address: Menzies Institute for Medical Research College of Health and Medicine University of Tasmania Hobart Tasmania Australia.; ^10^ Present address: School of Computing, Engineering and Mathematical Sciences La Trobe University Bundoora Victoria Australia.

**Keywords:** aerobiology, allergic rhinitis, allergy, evaluation, pollen monitoring

## Abstract

**Background:**

Globally, many pollen monitoring networks provide the community with daily pollen information, but there are limited data on health consumer uses and benefits. This research investigated why individuals in the community access pollen information, how they use it, and the perceived benefits.

**Methods:**

In‐ and post‐pollen season surveys (2017–2018 and 2018–2019) enquired about symptoms, diagnoses, symptom management, access, benefits and usefulness of pollen information provided by the AusPollen Partnership. Open text responses were examined by thematic analysis. Theme frequency and quantitative data were compared across pollen seasons, within and after the season, and between respondents with and without access to AusPollen information.

**Results:**

Surveys were completed 4044 times by 3604 individuals who predominantly self‐reported severe and frequent allergic rhinitis symptoms. Local AusPollen information was accessible to 84.6% of participants, and was reportedly used for preparation and planning (34.6%), guiding activities (32.9%), and medication decisions (28.2%). When asked how pollen information helped, similar themes were evident; but 16.1% also mentioned safety for themselves and others. However, secondary analysis of survey responses indicated that self‐reported medication use did not differ between those with or without access to pollen information or between time points surveyed. Suggestions for improvement included extended duration (16.4%), wider geographic range (13.5%), and information on other taxa (17.2%).

**Conclusion:**

There was a perceived need for localised, detailed and timely pollen information by people with pollen allergy. Whilst responses suggested this helped inform behaviours linked to allergen avoidance, further education strategies on allergic rhinitis control are needed to support patients who self‐manage their condition.

## 1 BACKGROUND

Many agencies or academic research groups worldwide monitor ambient pollen levels and provide local daily pollen information to the community, with considerable time, expertise and expense required to provide this service.^1^, ^2^ While there are multiple uses of pollen information, including clinical and environmental health, ecological and climate change research, the primary motivator for pollen monitoring is to assist people to manage their respiratory allergy. However, there is scant information on the ways in which respiratory allergy patients use pollen information, and the real‐world benefits of such information.

Up to 70% of allergic rhinoconjunctivitis (AR) sufferers self‐manage their condition with no health care provider input.^3^, ^4^ This situation leaves many people with AR with insufficient medical management of this condition, which means AR sufferers may be influenced by pharmaceutical marketing and purchase multiple products, and may struggle to find evidence‐based or personalised information on management of their conditions.^5^ Rather than the more effective preventers such as intranasal corticosteroids (INCS) that control underlying‐allergic inflammation, over‐the‐counter antihistamines are the most common means of self‐management of AR.^3^, ^6^, ^7^, ^8^ Unfortunately, leaving AR untreated is likewise common.^9^ Furthermore, uncontrolled AR has a deleterious impact on asthma, with a significant burden on public health systems.^10^, ^11^

The plethora of mobile health applications designed to improve health literacy and outcomes highlight the need for patient‐centred health management tools,^12^, ^13^, ^14^ and assume an appetite in the community for accessing health and environmental exposure information.^15^, ^16^ However, there is a need for further evidence that mobile health interventions are effective and provide a health benefit to consumers.^17^ The AusPollen Partnership in Australia sought to establish a standardised pollen monitoring network that would provide daily grass pollen information to the community during the relevant local grass pollen season.^18^ Four AusPollen Partnership research sites provided daily grass pollen information directly to community members using mobile phone apps and webpages for four in Eastern Australia cities: Brisbane, Sydney, Canberra and Melbourne during Austral pollen seasons from 2016 to 2020. This included the previous day's grass pollen concentration, and daily forecasts. Using an implementation science framework,^19^ the utility and benefit of providing this pollen information was investigated during the project by surveying individuals who have access to the pollen information in comparison with individuals in places where this information was not provided. A pilot evaluation questionnaire administered in the first year of the AusPollen partnership project surveyed how people use local current pollen information provided by webpages and smart phone apps.^20^ The pilot evaluation study collected and analysed data from a small number of users with and without access to daily local pollen information. Core themes expressed about why individuals access pollen information included allergic disease prevention, allergen avoidance, preparation and planning.^20^

This study extends the pilot study to evaluate how access to pollen information helps users, and further, whether access to pollen information supports users to better control hay fever and/or asthma. We also asked how the AusPollen, and potentially other pollen information services, might be improved to better meet the needs of community users with allergic respiratory diseases.

## 2 METHODS

### 2.1 Questionnaires

The study was approved by the South Western Sydney Local Health District Human Research Ethics Committee (HE16/158). The questionnaire was developed in consultation with AusPollen partner organisations including the peak professional body the Australasian Society for Clinical Immunology and Allergy, and the patient advocacy foundation Asthma Australia. The survey contained sections on demographics, respiratory allergy symptoms, symptom management, and evaluation of pollen information. Full details on survey elements are provided in Supporting Information S1, and Medek et al.^20^ The In‐season questionnaire was based on the pilot study, and contained additional and modified questions. Specifically, the In‐season questionnaire surveyed ethnicity, and asked about self‐reported rather than medically diagnosed hay fever. The symptom severity questions asked, ‘During the season when you experience symptoms, how often do your symptoms bother you?’. In the medication section, respondents were asked about asthma relievers and preventers separately. The questionnaire focussed on access to AusPollen pollen information rather than ‘pollen information’ in general. A Post‐season questionnaire included questions on the frequency of AusPollen information access, reason for access, how it was helpful, any other information people would want from the AusPollen Information service, and suggestions for improvement using the question ‘If you have used our AusPollen service, have you got any suggestions on how we can improve it?’

### 2.2 Survey distribution

Individuals in the community were invited to participate in both an In‐season and a Post‐season questionnaire over two seasons, both of which had less than average spring rainfall over the study area with the exception of the Brisbane area. The grass seasonal pollen index ranged from 515 to 3076 pollen/m^3^ across the two seasons for Sydney, Canberra and Melbourne, whilst in the subtropical site of Brisbane the grass seasonal pollen index was 10,512 and 9082 in seasons one and two, respectively.^18^ The In‐season questionnaire was open from 13 October 2017 to 9 March 2018 (first season) and 16 September 2018 to 2 November 2018 (second season). The Post‐season questionnaire was open from 16 January 2018 to 9 March 2018 (first season) and 30 January 2019 to 9 June 2019 (second season) to allow for the northern state of Queensland to have a pollen season extending until May, and thus a later start and end date.^18^

Participants were recruited from the community by newsletters distributed by the Australasian Society of Clinical Immunology and Allergy (ASCIA), Asthma Australia and the National Allergy Council, as well as links on the AusPollen websites. Adults who received information from these sources, and thus had a self‐selected interest in allergy, were eligible to participate. It was expected that those who chose to participate would have an allergic respiratory condition. Additionally, those who provided email addresses in previous surveys, including the pilot study, were directly emailed in subsequent survey rounds to invite them to complete the survey again. When individuals clicked the link to the questionnaire, they were informed about the study, and were asked to indicate their consent to participate by commencing the online survey. Participants were asked about their allergy diagnoses and symptoms if present. People under 18 years and respondents who lived outside Australia were excluded. Participants were allocated into groups with and without access to information from AusPollen sources.

### 2.3 Qualitative analyses

A reflexive thematic analysis^21^, ^22^ was performed using a combination of deductive and inductive qualitative analytical approaches based on the responses to the following open‐ended questions:

1. ‘How did the AusPollen count information help you?’ (Post‐season 2017, with access to AusPollen information)
2. ‘In what ways do you think the pollen count is useful to you?’ (In‐season 2017, In‐ and Post‐season 2018, with access to AusPollen information)
3. ‘Why did you access local pollen count and forecast information?’ (Post‐season both years, with access)
4. ‘Why would you want to access local pollen count and forecast information?’ (both years In‐ and Post‐season surveys, without access to AusPollen information)

#### 2.3.1 Inductive analysis

A random sample of 10% of responses was subjected to an initial coding and inductive thematic analysis by two members of the team (DM and JMD) to identify prevalent themes. Consensus on the themes was then reviewed with a third researcher (CHK). A second 10% resampling was taken from responses to each question and coded by DM, and reviewed together with JMD, to assess the consistency and refine codes and themes.

#### 2.3.2 Deductive analysis

A 90% sample (excluding those from the initial analyses) was obtained from the survey responses for deductive analysis. A list of words used exclusively in responses coded during the inductive analyses was produced for selected themes. These were supplemented with two‐word phrases where appropriate. For example, those coded ‘inside/outside’ produced a list including words such as ‘indoor’, ‘outdoor’, ‘go out’, ‘going out’, ‘went out’, ‘outing’, ‘be out’, ‘inside’, ‘outside’, ‘stayed’, ‘leaving’ and ‘venture out’. Words that did not appear to be related to the theme (in the above case, including ‘mitigate’, ‘nasal’, ‘regard’) were excluded. For words where uncertainty existed, and for two‐word phrases, instances of their use were retrieved from the inductive analysis sample and from the entire dataset, and they were included based on a high specificity and sensitivity for identifying responses related to the theme. The responses mentioning words from the word list were then retrieved and coded.

### 2.4 Statistics

Participants who responded to more than one survey (first and second season, In‐ and Post‐season) were identified. For quantitative data reported here, only the first survey response is included for any participant who responded more than once. Quantitative survey responses were analysed descriptively and when appropriate, differences in frequency of categorical responses analysed using chi squared tests in R (version 3.6.1, 2019‐07‐05).

## 3 RESULTS

### 3.1 General characteristics of survey respondents

There were a total of 4044 questionnaires submitted in the In‐ and Post‐season surveys across the two years (Table S1). These questionnaires were completed by 3604 participants, of whom 336 individuals provided multiple responses across the four surveys. A total of 3048 participants reported having access to AusPollen pollen information, and 503 reported not having access or did not know whether they had access (Table S1). Of those who did not have access, 85% stated they wanted access to local pollen count and forecast information (Table S1).

More participants were female in season 2 (66%) than season 1 (58%; Table 1). More participants were born in Australia in season 2 (76%) than in season 1 (67); and more participants in the second season were older than the first season (Table 1). Respondents, particularly those with access to pollen information, were concentrated geographically around and between Australia's eastern cities consistent with the location of AusPollen sites, whereas those without access to pollen information came from more regional locations and cities without AusPollen monitoring sites (Figure S1).

**TABLE 1:** Demographics of the survey respondents, and self‐reported allergic rhinitis and asthma.

	Season 1	Season 2	Chi square
Number of participants	2526	1078	
Gender (*n* = 3578)^a^			
Female	1449 (58%)	703 (67%)	*p* < 0.0001
Male	1053 (42%)	355 (33%)	
Other	12 (0.5%)	6 (0.6%)	
Born in Australia (*n* = 3577)			
Yes	1595 (67%)	774 (76%)	*p* < 0.0001
No	917 (33%)	291 (24%)	
Self‐reported AR (*n* = 3576)	2379 (95%)	1004 (94%)	*p* = 0.23
Self‐reported diagnosis of asthma (*n* = 3588)	1244 (49%)	634 (59%)	*p* < 0.0001
Both AR + asthma (*n* = 3588)	1176 (46.7%)	599 (56.0%)	*p* < 0.0001
Age in years (*n* = 3536)			
Age 18–25	114 (5%)	42 (4%)	*p* < 0.0001
Age 26–30	177 (7%)	58 (6%)	
Age 31–40	707 (30%)	201 (20%)	
Age 41–50	637 (27%)	266 (26%)	
Age 51–60	499 (21%)	206 (20%)	
Age over 60	238 (10%)	257 (25%)	

^a^ Overall number of respondents for each question.

### 3.2 Allergic rhinitis and asthma status of participants

As anticipated, respondents of surveys across both seasons self‐reported similarly high rates of AR (95% and 94% for the first and second survey, respectively), with 49% reporting a formal diagnosis of asthma in the first survey, and 59% in the second survey (Table 1).

**FIGURE 1:**
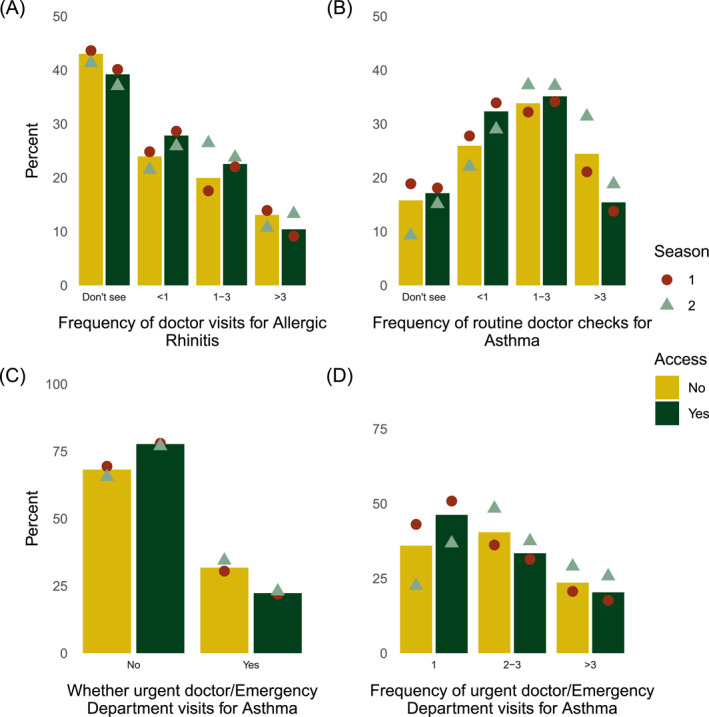
Self‐reported healthcare visits of participants with allergic rhinitis and asthma, as an average for those without (yellow bars) and with (green bars) access to pollen information, with the response for season 1 (red circle) and 2 (green triangle). (A) Of respondents who had AR (3383), percent who ‘don't see’, or see a doctor for AR various times a year. (B) Of respondents with self‐reported asthma (2017), percentage who ‘don't see’ or made a routine doctor visit, with frequency of visits. (C) Of respondents with self‐reported asthma (2136), percent who had an urgent visit for their asthma in the last year. (D) Of those who responded ‘Yes’ in (C) (502), percent who attended various times in the last year. AR, allergic rhinoconjunctivitis.

Of the respondents with AR, an average of 40% (37% to 44%, depending on season and access) indicated they did not see a doctor for their AR, whereas 27% (22%–28%) saw a doctor less than once a year, and 11% (9%–14%) saw a doctor for AR more than three times a year (Figure 1A). Of respondents who indicated they had been told by a doctor or nurse they had asthma, 17% (9%–19%, depending on season and access) reported they did not see a doctor for a routine asthma check‐up, whereas 31% (22%–34%) saw a doctor less than once per year for an asthma check‐up, and 17% (14%–31%, depending on season and access) saw a doctor more than 3 times a year (Figure 1B). Of the respondents with asthma, 24% (22%–34%, depending on season and access) visited a doctor or hospital urgently in the previous year for this condition (Figure 1C), and of those who did so, 44% (23%–51%) had one urgent visit, and 21% (18%–29%) had more than three urgent visits (Figure 1D).

**FIGURE 2:**
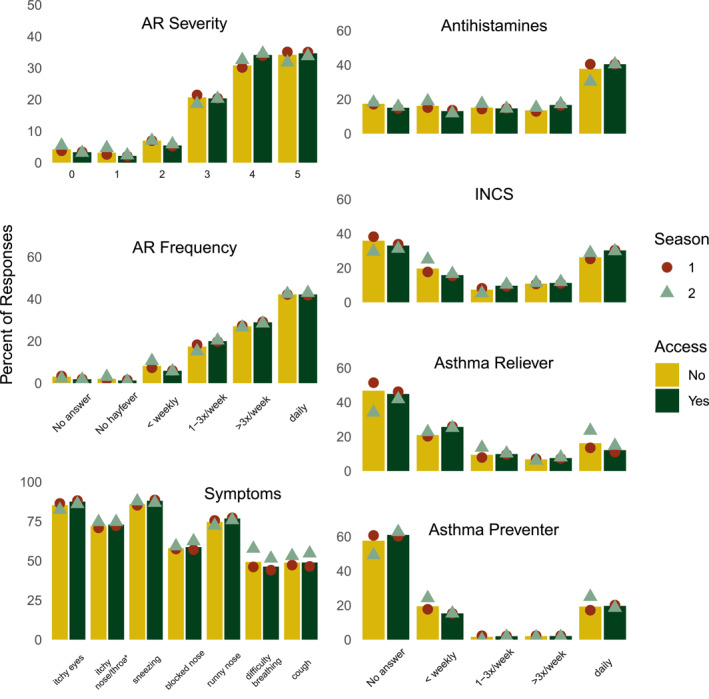
Self‐reported severity, frequency, and symptoms of self‐reported allergic rhinitis, and frequency of medications used including oral antihistamines, intranasal corticosteroids (INCS), inhaled asthma relievers and inhaled asthma preventers, in those without (yellow bars) and with (green bars) access to pollen information, with the response for the first (red circles) and second (green triangles) seasons. Bars represent the percentage of respondents for each question: AR severity, 3456, AR frequency, 3536, Symptoms, 3486, and medications used 3536. Respondents could select one answer to each question, except for ‘Symptoms’, for which multiple responses were allowed. AR, allergic rhinoconjunctivitis; INCS, intranasal corticosteroids.

Across both years, and between those with and without access to AusPollen information, most people rated their AR as moderate (3–4 out of 5 [54%]) or severe (5 out of 5 [34%]). Most participants reported experiencing symptoms more than three times a week or daily during the season in which they were most affected (Figure 2). Each of the symptoms asked about in the survey were reported to have been experienced by approximately 50% or more of the participants, with the commonest responses including itchy eyes, runny nose (rhinorrhoea) and sneezing (Figure 2).

Overall (*n* = 3604), more respondents reported using antihistamines (39.8% daily) than INCS (29.6% daily, *p* < 0.0001); however, fewer reported using asthma relievers (12.8%) than asthma preventers (19.7%, *p* < 0.0001; Figure 2). There were no apparent differences in symptoms or medication use between seasons or between those who did and did not have access to AusPollen information (Figure 2).

### 3.3 Where people accessed information about hay fever

**FIGURE 3:**
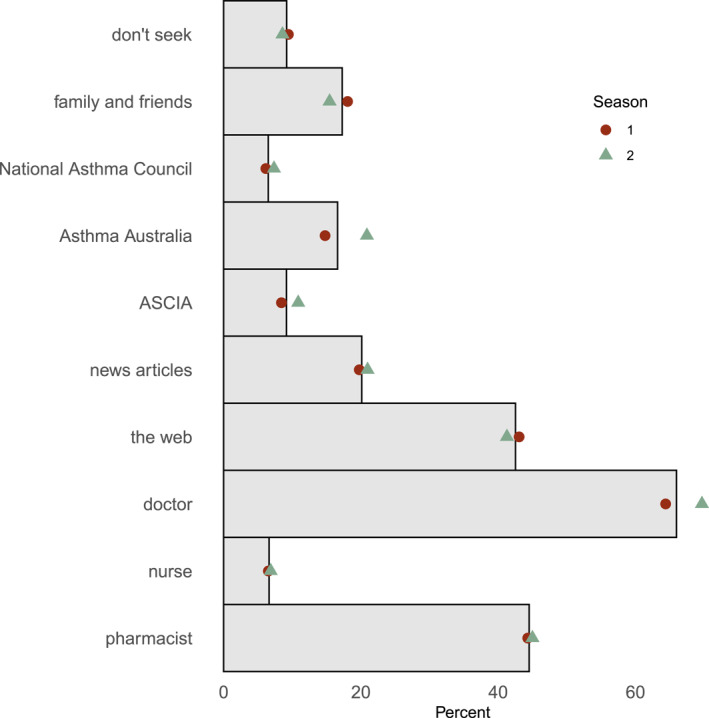
Responses to the multiple‐answer question about sources of hay fever information. Respondents (*n* = 3518) could select more than one option. Bars reflect percentage of total responses, with points reflecting season one (red circle) and season two (green triangle). ASCIA, Australasian Society of Clinical Immunology and Allergy.

The majority of participants reported obtaining information on their hay fever from their doctor (66.1%), the Web (42.4%), or a pharmacist (44.6%). Few (9.2%) stated they did not seek information on their hay fever. Information seeking patterns differed between years of the survey (Figure 3). More respondents obtained information from Asthma Australia, the primary non‐government organisation providing support to asthma patients, the ASCIA, the peak professional body for clinical immunologists and a primary source for patient and carer allergy information, and their doctor in the second season than the first season (Figure 3, *p* = 0.002).

### 3.4 Reasons for access and uses of pollen information

**FIGURE 4:**
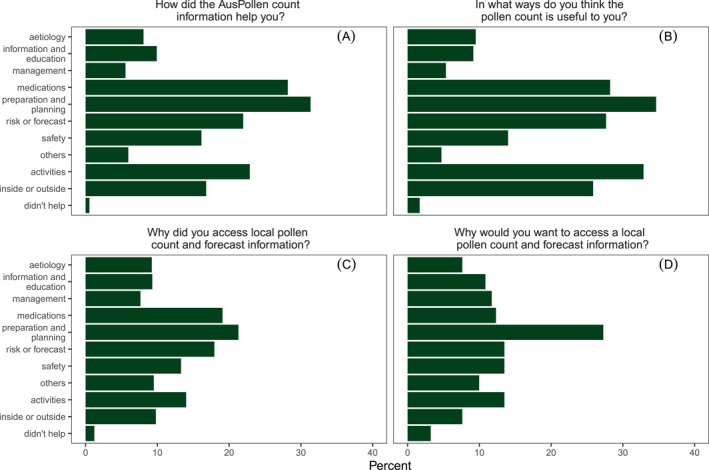
Thematic analysis of how pollen information was (A) helpful or (B) useful, and (C) why it was accessed (in those with access) or (D) why access was wanted (in those without access). Bars represent percentage of responses coded to each theme from a total (A) 756, (B) 1779, (C) 1399, and (D) 341 responses. Deductive analysis of 90% of responses is presented. Inductive analysis of the initial 10% is presented in Figure S3.

Cross‐cutting themes were identified following a review of initial codes extracted from the open‐answer questions regarding pollen information uses (Figure S2), relating to how it was helpful and why it was accessed or would be accessed. Salient themes with proportions of total responses mentioning each theme are presented in Figure 4. Overall, similar trends existed across inductive analyses (Figure S3) and deductive analyses (Figure 4), however ‘information and education’ appeared more frequently in the inductive analysis than in the deductive analysis.

#### 3.4.1 Risk or forecast

Respondents reported that pollen information communicated to them the risk of pollen allergy, or that they wanted to know the pollen forecast.

‘It tells me when the pollen count is likely to be high’ (Post‐season 2 response 109, Caucasian female, 51–60). The greatest proportion of responses with this theme were in answer to, ‘In what ways do you think the pollen count is useful to you?’ (27.7%).

#### 3.4.2 Preparation and planning

A large proportion of respondents used these and similar words; ‘To make sure I am prepared’ (Post‐season 1 response 908, Asian female, over 60). The greatest proportion of responses coded to this theme was in response to the question, ‘In what ways do you think the pollen count is useful to you?’ (34.6%).

#### 3.4.3 Activities

Respondents described that the pollen information was useful to guide actions and activities, indicating that pollen information would guide behaviour; ‘We knew on high pollen count days to keep windows closed where possible!’ (Post‐season 1 response 690, Caucasian female, 51–60), and ‘Knew when it was the best for walking’ (Post‐season 1 response 75, Caucasian female over 60). The greatest proportion of responses coded to this theme, 32.9%, were in response to ‘In what ways do you think the pollen count is useful to you?’

#### 3.4.4 Inside or outside

As a subset of the theme on activities, most responses described decisions regarding going outdoors or staying inside; ‘Helped me decide if I could go outside during the day’ (Post‐season 1 response 874, Caucasian female, 26–30). There were 25.9% of responses to ‘In what ways do you think the pollen count is useful to you?’ coded to this theme.

#### 3.4.5 Aetiology

Some respondents described how pollen information provided insights into the causes of their symptoms. ‘There is correlation but I don't think pollen is the main cause of my hay fever’ (In‐season 1 response 412, Caucasian male, 41–50), and ‘Confirms if my symptoms are related to hay fever’ (In‐season 1 response 581, Asian male, 31–40). Up to 9.4% of responses coded to this theme, the commonest being in response to, ‘How did the AusPollen count information help you?’

#### 3.4.6 Information and education

Some respondents appreciated the pollen information, either for information's sake or to become more aware. Several used the information to educate others; ‘Education of patients’ (Post‐season 1 response 1068, Caucasian male, over 60), and ‘Helps build up information to control my symptoms’ (Post‐season 1 response 214, Caucasian male, over 60). There were up to 10.9% of responses coded to this theme, with the highest proportion being in those without pollen information responding to ‘Why would you want to access local pollen count and forecast information?’

#### 3.4.7 Understanding

As a subset of the information and education theme, respondents sought to understand their symptoms, risk of flares up and what made symptoms worse. ‘To try and understand why some days were better than others’ (Post‐season 2 response 761, Caucasian female, 51–60). Understanding was most frequently mentioned in response to the question, ‘How did the AusPollen count information help you?’, at 1.3% of responses.

#### 3.4.8 Awareness

Awareness was another subset of the information and education theme. Patients became more aware of pollen levels, and risks, for instance, going outdoors; ‘Awareness of pollen and what I could do that is, garden work on low pollen days’ (Post‐season 2 response 898, Caucasian female, 26–30), and ‘I'm made aware of high pollen count and try not to be out much’ (In‐season 1 response 990, Caucasian female, 41–50). However, responses mentioning awareness were often more general; ‘keep me aware’ (Post‐season 2017 response 566, Female, 31–40, ethnicity not disclosed). Awareness was mentioned most often in response to the question, ‘In what ways do you think the pollen count is useful to you?’ at 4% of responses.

#### 3.4.9 Management

A theme of symptom or disease management was evident in up to 11.7% of responses, ‘Helped me to manage my allergies and asthma better’ (Post‐season 1 response 537, Caucasian female, 26–30), the most being in those without access to pollen information, responding to, ‘Why would you want to access (a) local pollen count and forecast information?’

#### 3.4.10 Safety

Concerns were expressed about the safety of the respondent, or of those they care for, using words associated with safety such as ‘warning’, ‘alert’, or discussing the symptoms or care of others; ‘Stay informed and on alert’ (Post‐season 1 response 333, Caucasian female, 41–50), and ‘Warned for future attacks’ (Post‐season 1 response 271, Caucasian male, 41–50). There were up to 16.1% of responses coded to this theme, with the greatest proportion being in response to the question, ‘How did the AusPollen count information help you?’

#### 3.4.11 Other people

Within those coded ‘safety’, responses specifically discussed the care of others; ‘I could use it to warn and protect my parents’ (Post‐season 1 response 70, Indigenous Australian male, 41–50), ‘My 12 years old son also has hay fever and gets asthma due to the hay fever. I use the pollen count mainly for him as advised by the [hospital], and give him an extra dose of antihistamine if the count is high or extreme to assist in preventing an asthma attack’ (In‐season 1 response 315, European female, 31–40). There were up to 9.9% of responses coded to this theme, the most being in those without access to pollen information, responding to, ‘Why would you want to access (a) local pollen count and forecast information?’

#### 3.4.12 Medications

Comments regarding medications were a common theme, for example, ‘I can anticipate and take a tablet or nasal spray if going for a day outdoors’ (In‐season 2 response 88, Caucasian female, 41–50). Approximately one third of responses regarding how the pollen information was useful, or how it helped (both 28.2%), mentioned medications. Interestingly, many respondents used pollen information to decide when to take antihistamines; ‘The pollen count helped me to decide whether to take an antihistamine tablet for that day’ (Post‐season 2 response 452, Caucasian male, older than 60); ‘Knowing the forecast pollen count helps me decide whether to take hay fever medication in advance, rather than waiting for symptoms to occur, by which time the medication doesn't seem to be as effective’ (In‐season 1 response 1154, Caucasian male, 41–50); ‘Count = low, medication = no!:‐)’ (Post‐season 1 response 995, Caucasian male, 51–60). Antihistamines were mentioned by 10.5% of responses regarding how the pollen information was useful. Nasal sprays were mentioned by 2.0%, and inhalers were mentioned by 3.4%. Interestingly, while other forms of treatment were mentioned less, the frequency of mentions of inhalers was higher at 4.6% in those responding to why they accessed pollen information in the Post‐season survey. We assessed for positive and negative valence in responses, by searching for words such as ‘bad’, ‘severe’, ‘suffer’; and ‘good’, ‘glad’, and ‘relief’. While the phrase ‘on bad days’ was used frequently, pollen information appeared on the whole to be relieving rather than contributing to the respondent's anxiety; ‘I could take an antihistamine at the sign of first symptom if the forecast was high pollen and I felt more relaxed prepared and symptoms under control. Without the info I used to wait until symptoms got worse’ (Post‐season 2 response 1050, Caucasian female, 41–50). Negative valence predominated (greatest at 17% in response to ‘Why did you access local pollen count and forecast information?’), with positive valence being highest (2.8%) in response to ‘In what ways do you think the pollen count is useful to you?’

Anxiety about thunderstorm asthma seemed to prompt several people to access pollen information; ‘have bad allergy and scared of thunderstorm asthma’ (Post‐season 2 response 566, Female, 41–50, ethnicity not disclosed), and ‘To see if I'm going to die’ (Post‐season 1 response 309, Caucasian male, 31–40). The highest proportion mentioning thunderstorms was in response to the question, ‘How did the AusPollen count information help you?’ (3.8%).

#### 3.4.13 Didn't help

Those who did not find pollen information helpful or useful explained that this was due to information not being provided for their location, not providing information on pollen they were allergic to, not being provided at the time they experienced symptoms, or not matching their symptoms. ‘Only some use because Melbourne and Churchill (a regional town) data did not reflect local conditions’ (Post‐season 1 response 800, Caucasian male, over 60); ‘It's not useful to score one area for whole city’ (In‐season 2 response 162, Subcontinental Indian male, 31–40), ‘I'm bad with some tree pollens, Canberra Pollen seems to only give info on grass pollens so it isn't as useful as it could be’ (In‐season 2 response 124, Caucasian female, 41–50). There were up to 7.9% of responses manually coded to this theme from the inductive method, with the highest proportion being in the answer to ‘In what ways do you think the pollen count is useful to you?’. Smaller proportions (up to 3.2%) were coded to this theme using the deductive method, but this method appeared less specific and may also have been less sensitive for this theme.

### 3.5 Other information wanted and how to improve pollen monitoring information

**FIGURE 5:**
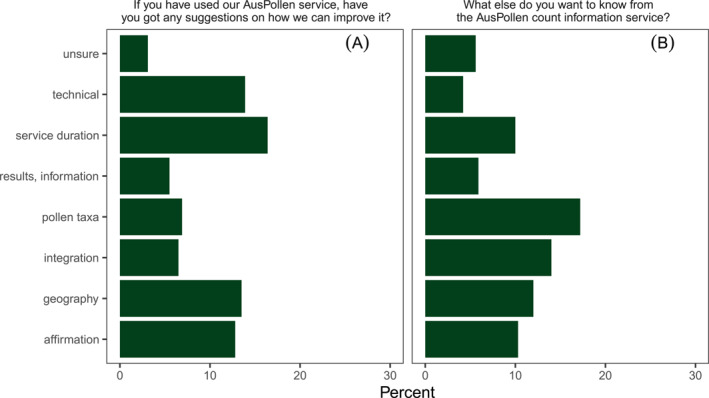
Responses to the open‐ended questions on how the pollen information could be improved or expanded. Results of deductive analyses are presented from a total of (A) 890 and (B) 827 responses analysed.

In the first and second Post‐season survey, 625 and 440, respectively, answered the question on how the AusPollen information could be improved (Figure 5). Similarly, for these Post‐season surveys 859 and 407, respectively, answered the question, ‘What else do you want to know from the AusPollen count information service?’ To these questions, responses followed the following themes:

#### 3.5.1 Service duration

Respondents suggested extending the duration of the pollen information, ‘It would be nice if it started earlier as my symptoms are often worst in September’ (Post season 2 response 91, Caucasian male, 41–50). Some (16.4%) of respondents wanted longer service duration when responding to the question, ‘If you have used our AusPollen service have you got any suggestions on how we can improve it?’

#### 3.5.2 Pollen taxa

17.2% of respondents wanted information on other pollen taxa in response to ‘What else do you want to know from the AusPollen count information service?’, for example, ‘Provide more details about the levels of non‐grass pollen’ (Post season 1 response 169, Caucasian male, 41–50).

#### 3.5.3 Integration

Fourteen percent of respondents wanted integration of pollen information with existing services such as air quality and meteorological information, health information and maps, ‘Integrate pollen forecast with existing weather apps like BoM’ (Post season 1 response 747, Asian male, 31–40).

#### 3.5.4 Information and education

Sixteen percent of respondents wanted detailed information and further education on pollen, ‘Enable access to historical pollen counts and personal input. It would be good to see and compare between seasons to correlate with treatment options’ (Post season 1 response 589, Asian male, 41–50).

#### 3.5.5 Geography

13.5% of respondents suggested finer‐grained geographic information. ‘More pollen collection stations in the Australian Capital Territory. The variety of wild grasses and differences in open spaces and proximity to cleared land versus bushland’ (Post season 2 response 872, Caucasian male, 41–50).

#### 3.5.6 Affirmation

12.8% of respondents had no further suggestions on how to improve the pollen information, responding with positive messages affirming the utility of the AusPollen apps: ‘Keep doing a good job’ (Post season 1 response 395, Caucasian male, 18–25).

#### 3.5.7 Unsure

5.6% of respondents had no further suggestions, and were unsure how to improve the service, ‘Not sure’ (Post season 2 response 176, Caucasian female, 31–40).

#### 3.5.8 Technical

13.9% of respondents provided suggestions regarding technical improvements in presentation or function of the AusPollen app: ‘A better interface. The graph data is squashing all the info and can make it hard to read’ (Post season 2 response 692, Asian male, 31–40).

## 4 DISCUSSION

Whilst it is often assumed and asserted that providing local daily pollen information and forecasts are useful, there are few studies purposefully designed to understand why health consumers seek pollen information, and how they use this information. In this study, we discovered an array of reasons why people in the community want access to pollen information, some of which are heuristic in nature (for information, awareness, and understanding), whereas others reasons were more practical, such as being a basis to guide decisions on medication, activities and behaviours for themselves and people they care for (e.g., children with asthma).

The cohort was over‐represented with participants who reported having a diagnosis of asthma, which was four to five times higher than the general rate of asthma of 11.2% in the Australian population.^23^, ^24^ However, the prevalence of asthma in AR patients in Australia is higher than in the general population.^25^ Participation in this study by people with asthma may have been influenced by the epidemic thunderstorm asthma event in Melbourne the previous spring (21 November 2016).^26^ This is consistent with the increase in the number of people accessing information specifically from the MelbournePollen.com.au site during the weeks following the thunderstorm asthma event.^27^ Importantly, some of these respondents reported fear and anxiety, which has subsequently been examined in adolescents with asthma.^28^ This theme, and the high representation of individuals reporting asthma, may have been influenced by an information bias due to prevalent media attention, heightened community awareness, and implementation of a number of public health responses in the state of Victoria including regional expansion of the pollen monitoring network, and a pollen and thunderstorm asthma forecast and alert programme,^29^ as well as use of the relevant Melbourne Pollen app at the time.^27^

Based on the proportion of people with hay fever who consult a doctor about their condition reported in previous studies,^3^, ^4^, ^8^ the number of participants reporting information from a doctor seemed higher than expected. However, in a separate study of influences on people with hay fever, those with more severe AR symptoms tended to seek advice from general practitioners more often than those with less severe AR (27). Whilst the severity of AR reported by the study participants was not confirmed objectively, our results suggest that about a third of respondents in this study may be in this more severe category, which would at least in part account for the higher than expected rate of doctor engagement.^30^

Participants were seeking knowledge, that is, information, awareness, and understanding, to explain the aetiology of their disease and to weigh up risks based on the pollen information and forecasts. Whether this knowledge led to empowerment that translated into behaviour change is difficult to gauge with this type of study design, but it has been effective in other settings.^4^, ^31^ However, the translation of knowledge into behaviour change was suggested by some of the other expressed themes: planning, preparedness and medication use. Whilst we could not test for changes in activities such as staying indoors, there were concrete examples provided to this effect that should lead to improved control of hay fever. We note that at the time of implementation, the purpose of the AusPollen Partnership was to establish a standardised national pollen monitoring programme^18^, ^32^ rather than to advise on or detect changes in medication use. Whilst the study was not designed or powered to detect change in behaviour, in secondary analysis, participant reported medication use did not appear to differ within and after the season, and between those with and without access. This is reflected in the reported variability of medication use patterns that pollen information prompted, ranging from being reminded to take medications to avoiding medications when pollen indices were low. It is important to highlight that whilst clearly helpful and beneficial to patients in the community, the use of information on pollen exposure to underpin allergen avoidance or medication use strategies does not on its own address the ongoing unmet need of many AR patients to access appropriate medical care.^3^, ^4^

It is also important to emphasise that the reported AR (and asthma) medications used were predominantly symptom relievers, whereas international guidelines recommend preventative medications as the mainstay of therapy.^33^ This was additionally expressed in the themes of reasons why people wanted access to pollen information and how they used that information. Whilst this is consistent with other literature on patient reliance on symptomatic relief over INCS in AR,^34^ it is nonetheless cause for concern. Indeed, some respondents requested further information on ‘debunking myths’ about medications. People sought pollen information for valid reasons but seemed to lack knowledge on effective medication strategies; despite reporting moderate (54%) or severe symptoms (34%), very few (29% daily, and 12.4% three or more days per week) reported the use of INCS to control inflammation. These data further indicate a need to better educate, even people with frequent severe symptoms, on how to best control hay fever. Further, one could question whether watching daily pollen information and forecasts encourages reliance on the use of symptom relievers over inflammation control medication.

Although we specified the questions related to access to ‘AusPollen pollen information’, we cannot rule out access to pollen information from other sources. Apart from AusPollen sites, and pollen monitoring in Tasmania by AirRater collaborators,^35^ other publicly available pollen forecasts in Australia, at the time of this study, may have come from unsubstantiated sources in places where pollen was not monitored or in places where advertising or other apps publish unsubstantiated forecasts.^36^ Such unsubstantiated and unvalidated pollen forecasts that are not informed by actual pollen monitoring data may provide misinformation to AR patients, carers, clinicians, and other stakeholders, which is a cause for concern. Although provision of pollen information per se may not be considered a mobile health (mhealth) application, national^12^ and international guidelines^13^ require mHealth apps to deliver benefit to patients.

A limitation of this study is that some modifications were made to the questionnaires between the first and second seasons. These were introduced to more specifically refer to AusPollen as opposed to other (unverified) pollen information for the aforementioned reason, to ask about perceived symptoms not only hay fever symptoms, and to use the same wording in‐ and post season for the question relating to how people used pollen information. However, these changes that were at the time considered valuable to enhance clarity potentially introduced changes in how respondents perceived these questions, which made direct comparison between years challenging. To address this issue, the survey questions in Figure 4A,B were analysed separately, and were found to give rise to similar explanatory themes about how access to pollen information was useful and helpful. A further limitation was that there was no direct contact with eligible participants in the community, and thus we could not accurately gauge the survey response rate.

Whilst other reports articulate that pollen information is useful to health consumers,^37^ this study is novel and important in that it provides for the first time direct evidence and nuanced insight to why pollen information is accessed and used, and the ways in which it was felt to be useful by people in the community. The data clearly indicate that people with AR, and asthma, want to know pollen information, and appear to benefit from the reassurance that information provides.

Strengths of this study include the unique approach to directly survey and evaluate evidence for why people access pollen information, enabling consumer‐informed responses to perceived needs for pollen information and how service can be improved. The dataset included a large number of individuals from across the community in a country with high rates of hay fever,^38^ with rich open answer responses and quantitative data from the respondents. The thorough inductive and deductive analysis of such a large qualitative dataset provided rich understanding of how participants used pollen information, thereby providing deep insights into how to better assist respiratory allergy self‐management. Respiratory allergy patients do not appear to have their needs adequately met from information sources available.

Some participants wanted access to historical data so they can track their own symptoms in relation to pollen exposure, which would be consistent with inclusion of pollen information as part of digital health solutions to inform person‐centred care.^39^ There was a desire for pollen information to be integrated with weather and air quality messaging, broader geographical coverage, and for more frequent sub daily information. The latter could only be readily achieved by implementation of automated pollen monitoring,^40^ yet broader geographic coverage on this large continent is difficult to achieve by either Hirst‐style sampling and manual counting due to labour costs, or automated devices given the diversity of pollen types in biogeographically distinct regions,^41^ and high cost of sophisticated automated devices. Feedback on what other information the participants wanted to know, and how this service can be improved, illustrates the need for a more sophisticated design of integrated pollen information and apps specifically created to modify behaviour to achieve better AR and asthma control.^31^, ^42^ Examples of other Apps include AirRater that integrates air quality and pollen,^35^ and the Mobile Airways Sentinel Network‐AIR that has a focus on increasing adherence to medication use.^43^

Whilst helping allergy patients is a core motivation for monitoring, pollen information is used in tracking ecological response to climate change,^44^, ^45^ to profile airborne pollen diversity,^46^, ^47^, ^48^ track invasive species spread,^49^ in clinical trial analysis, epidemiology,^50^, ^51^ examination of thresholds of exposure associated with symptoms,^52^, ^53^ and integration with gaseous and particulate pollutants.^54^ The cost‐benefit analysis of generating and sustaining pollen information should encompass the full range of uses of pollen information^2^; by patients in the community, environmental health, ecological research uses, and interpretation of clinical immunotherapy trials in the context of exposure to triggers.^55^, ^56^

This study demonstrates that consumers in the community engaged with the pollen information on multiple occasions throughout the pollen season, and integrated it into their daily routine, thereby deriving multiple benefits. There was a perceived need for localised, detailed and timely pollen information by people with pollen allergy. Whilst responses suggested this helped inform behaviours linked to allergen avoidance, it did not appear to directly translate into appropriate medication use and adherence. This may indicate that respiratory allergy patients and carers in the community require additional support to encourage behaviour change related to medication usage. This could include additional educational resources, or active prompting/engagement for optimal management of AR. These findings emphasise that pollen information providers have a duty of care to provide accurate information, as well as advice on how to interpret and use the information.

## AUTHOR CONTRIBUTIONS

**Danielle E. Medek**: Conceptualization; funding acquisition; investigation; formal analysis; data curation; visualization; writing—original draft. **Constance H. Katelaris**: Conceptualization; funding acquisition; investigation; formal analysis; validation; writing—review & editing. **Andelija Milic**: Project administration; data curation; investigation; writing—review & editing. **Paul J. Beggs**: Conceptualization; investigation; funding acquisition; formal analysis; writing—review & editing. **Edwin R. Lampugnani**: Conceptualization; investigation; writing—review & editing. **Don Vicendese**: Methodology; investigation; writing—review & editing. **Bircan Erbas**: Conceptualization; investigation; funding acquisition; formal analysis; writing—review & editing. **Janet M. Davies**: Conceptualization; investigation; funding acquisition; formal analysis; project administration; supervision; writing—original draft; writing—review & editing.

## CONFLICT OF INTEREST STATEMENT

DM, CHK, PJB, BE and JMD received a grant from NHMRC, the AusPollen Partnership Project GNT1116107, with cash and in kind contributions from partner organisations, Australasian Society of Clinical Immunology Allergy, Asthma Australia; Stallergenes Australia; Bureau Meteorology, Commonwealth Scientific Industrial Research Organisation, Federal Office of Climate and Meteorology Switzerland, for this project.

JMD receives funding from the ARC, NHMRC, Department of Health and Ageing, and MRFF for research outside the scope of this study. Outside this research, JMD has conducted research in collaboration with Abionic SA, Switzerland, supported by competitive grant from the National Foundation for Medical Research Innovation with co‐contribution from Abionic. QUT has received support for other allergy research of JMD via in kind services or materials from Sullivan Nicolaides Pathology (Queensland), Abacus Dx (Australia), Stallergenes (France), Stallergenes Greer (Australia), Swisens (Switzerland), Kenelec (Australia), and ThermoFisher (Sweden). QUT owns patents relevant to grass pollen allergy diagnosis (US PTO 14/311,944 issued, AU2008/316,301 issued) for which JMD is an inventor. ERL is an Executive Director of AirHealth Pty Ltd Australia. Other authors declare no other competing interests.

## Supporting information

Supporting Information S1

## Data Availability

The data from this study is not available for sharing for privacy reasons because it may contain personal identifying information. Consent to share study data was not obtained from participants and approval to share data from this study was not granted by the Human Research Ethics Committee.
