# Supplementary material for: Aerobiology matters: Why people in the community access pollen information and how they use it
Source: Clin Transl Allergy. 2025 Jan 24;15(1):e70031. doi: 10.1002/clt2.70031 (PMC11761002; doi:10.1002/clt2.70031)
Supplement: Supplementary file 1 — Supporting Information S1 [file CLT2-15-e70031-s001.docx]

**Aerobiology matters: evaluation of why people in the community access pollen information and how they use it**

*Short title:*

Why and how health consumers use pollen information

Danielle E. Medek^1^, Constance H. Katelaris^2,3^, Paul J. Beggs^4^, Andelija Milic^1^, Edwin R. Lampugnan^5^, Don Vicendese^6^, Bircan Erbas^7^, Janet M. Davies^1^

*Clinical and Translational Allergy*

1 School of Biomedical Sciences, Centre Immunity and Infection Control, Centre for Environment, Queensland University of Technology, Kelvin Grove Queensland 4059, Australia

2 School of Medicine, Western Sydney University, Sydney, New South Wales 2751, Australia

3 Department of Immunology, Campbelltown Hospital, Campbelltown Sydney, New South Wales 2751, Australia

4 Department of Earth and Environmental Sciences, Faculty of Science and Engineering, Macquarie University, Sydney, New South Wales 2109, Australia

5 School of Biosciences, The University of Melbourne, Parkville Victoria 3010, Australia

6 The Melbourne School of Population and Global Health, University of Melbourne, Parkville Victoria 3010, Australia.

7 School of Public Health, LaTrobe University, Bundoora, Victoria 3086, Australia

**Corresponding author:**

Professor Janet Davies, Centre Immunology and Infection Control, Queensland University of Technology (QUT), Brisbane, Australia

Email: [j36.davies@qut.edu.au](mailto:j36.davies@qut.edu.au)

**Current affiliations**

Edwin R. Lampugnani, AirHealth Pty Ltd, Parkville Victoria 3010, Australia, and Menzies Institute for Medical Research, College of Health and Medicine, University of Tasmania, Hobart, TAS 7001, Australia. Dr Don Vicendese, School of Computing, Engineering and Mathematical Sciences La Trobe University, Bundoora, Victoria 3086, Australia.

# Supplemental Material

**QUESTIONS SHARED ACROSS PRE- AND POST- POLLEN SEASON QUESTIONNAIRES**

About you (Demographics)

Identifier

How old are you?

What is your gender?

Were you born in Australia?

How many years have you lived in Australia?

What is your ethnicity?

What is your postcode?

About your symptoms

Do you experience hay fever?

Has a doctor (or nurse) ever said you have asthma?

If you experience hay fever how severe does it get?

What makes your symptoms worse?

What symptoms do you get? (please choose all that apply)

What time(s) of the year are you affected by your symptoms? (please choose all that apply)

During the season when you experience symptoms how often do your symptoms bother you?

If you experience asthma does having hay fever at the same time make your asthma symptoms worse?

Controlling symptoms

Do you use any of the following treatments? (please select each you use)

Have you had allergen immunotherapy (regular desensitising drops or injections)?

What allergy or allergies was it for?

How often do you see a doctor for hay fever?

How often do you see a doctor for a routine check on your asthma?

Have you had an urgent visit to a doctor or hospital for your asthma in the last year?

How often did you visit?

Where do you get information on your allergies from? (Please check all that apply)

Would a local AusPollen App help?

(Do/ Did) you have access to a local AusPollen pollen count information (e.g. BrisbanePollen, CanberraPollen, MelbournePollen or SydneyPollen)?

Would you want to access a local pollen count and forecast information?

Why would you want to access local pollen count and forecast information?

In what ways do you think the pollen count information is useful to you?

**Questions asked in the Post-season Questionnaire only**

Access and use of AusPollen information

Did you complete the AusPollen pre-season evaluation questionnaire?

How often did you access pollen count information during the season you had symptoms?

Usefulness of AusPollen information

Why did you access local pollen count and forecast information?
Was the AusPollen pollen count information helpful?
How did the AusPollen count information help you?

Suggestions for improvement

What else do you want to know from the AusPollen count information service?

If you have used our AusPollen service, then have you got any suggestions on how we can improve it?

**SUPPLEMENTAL TABLE 1**. Total number of responses received to each of the surveys, and specific questions within these.

| **Pre and post season AusPollen Questionnaire respondents** | **Pre season 1** | **Post season 1** | **Pre season 2** | **Post season 2** | **Total number** |
| --- | --- | --- | --- | --- | --- |
| Total responses (percent of total) | 1,469 (36.3%) | 1,167 (28.9%) | 606 (15.0%) | 802 (19.8%) | 4044 |
| Total responses, unique individuals | 1,464 (40.6%) | 1,062 (29.5%) | 388 (10.8%) | 690 (19.1%) | 3604 |
| Have access to AusPollen information | 1,166 (33.6%) | 1,073 (30.9%) | 530 (15.3%) | 699 (20.2%) | 3468 |
| No access to AusPollen information | 298 (59.2%) | 60 (11.9%) | 69 (13.7%) | 76 (15.1%) | 503 |
| “I don't know” if have AusPollen information, post-season surveys | 0 | 32 (59.3%) | 0 | 22 (40.7%) | 54 |
| If had access, how many responded "In what ways do you think the pollen count is useful to you?" | 941  (46.7%) |  | 437 (21.7%) | 637 (31.6%) | 2015 |
| If had access, how many responded "Why did you access local pollen count and forecast information?" |  | 1,013 (60.1%) |  | 673 (39.9%) | 1686 |
| If access, how many responded "How did the AusPollen count information help you?" |  | 933 |  |  | 933 |
| If no access pre and post (or “I don't know”), how many wanted access | 248  (58.1%) | 52  (12.2%) | 56  (13.1%) | 71  (16.6%) | 427 |
| Percentage without access who wanted access | 83% | 87% | 81% | 93% | 85% |
| If no access, how many responded “Why would you want to access local pollen count and forecast information?” | 205 (55.3%) | 49 (13.2%) | 49 (13.2%) | 68 (18.3%) | 371 |


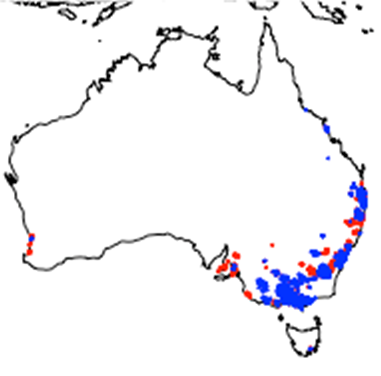


Red: respondents without access to AusPollen information

Blue: respondents with access to AusPollen information

**SUPPLEMENTARY FIGURE 1** Location of respondents


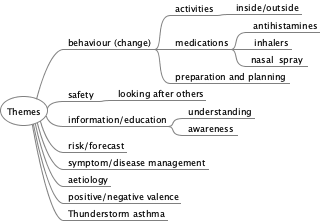


**SUPPLEMENTARY FIGURE 2** Initial codes identified in initial inductive thematic analysis of a 10% sample of responses.


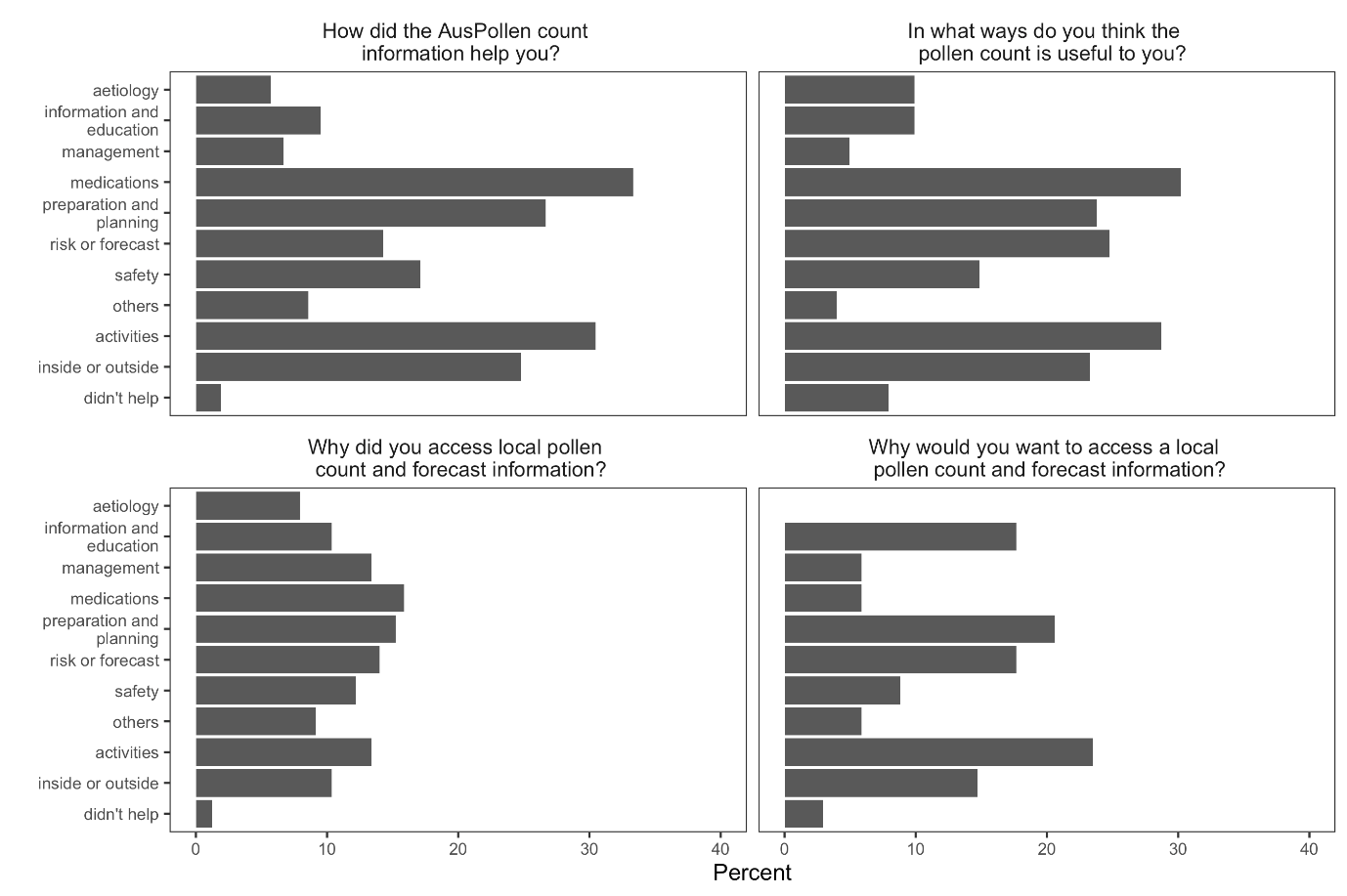


**SUPPLEMENTARY FIGURE 3.** Inductive thematic analysis of a second 10% random resampling of responses showing the percent of responses that expressed particular themes.
